# Puerarin Alleviates Blood Pressure via Inhibition of ROS/TLR4/NLRP3 Inflammasome Signaling Pathway in the Hypothalamic Paraventricular Nucleus of Salt-Induced Prehypertensive Rats

**DOI:** 10.3390/nu16162580

**Published:** 2024-08-06

**Authors:** Hong-Li Gao, Yu Yang, Hua Tian, Shen-Liang Xu, Bo-Wen Li, Li-Yan Fu, Kai-Li Liu, Xiao-Lian Shi, Yu-Ming Kang, Xiao-Jing Yu

**Affiliations:** 1 Department of Physiology and Pathophysiology, School of Basic Medical Sciences, Xi’an Jiaotong University Health Science Center, Xi’an 710061, China; happygaohongli@163.com (H.-L.G.); yangyu001@stu.xjtu.edu.cn (Y.Y.); 18092090577@163.com (H.T.); 18997279283@163.com (S.-L.X.); m17791211554_1@163.com (B.-W.L.); fuly4525@163.com (L.-Y.F.); kaililiu1115@163.com (K.-L.L.); shxl@mail.xjtu.edu.cn (X.-L.S.); ykang@mail.xjtu.edu.cn (Y.-M.K.); 2 Institute of Cardiovascular Sciences, Translational Medicine Institute, Xi’an Jiaotong University Health Science Center, Xi’an 710061, China; 3 Key Laboratory of Environment and Genes Related to Diseases, Xi’an Jiaotong University, Ministry of Education, Xi’an 710061, China; 4 Department of Pharmacology, Basic Medical College, Jiamusi University, Jiamusi 154007, China; 5 Department of Diagnosis, Shaanxi University of Chinese Medicine, Xi’an 712046, China; 6 Department of Pharmacology, School of Basic Medical Sciences, Xi’an Jiaotong University Health Science Center, Xi’an 710061, China

**Keywords:** puerarin, salt-induced prehypertension, ROS/TLR4/NLRP3 inflammasome signaling pathway, hypothalamic paraventricular nucleus, proinflammatory cytokines

## Abstract

Background: Puerarin is an isoflavone compound isolated from the roots of a leguminous plant, the wild kudzu. Various functional activities of this compound in multiple diseases have been reported. However, the effect and mechanism of puerarin in improving blood pressure remain non-elucidated. Purpose: The current study was designed to assess the preventive effects of puerarin on the onset and progression of hypertension and to verify the hypothesis that puerarin alleviates blood pressure by inhibiting the ROS/TLR4/NLRP3 inflammasome signaling pathway in the hypothalamic paraventricular nucleus (PVN) of salt-induced prehypertensive rats. Methods: Male Dahl salt-sensitive rats were fed low NaCl salt (3% in drinking water) for the control (NS) group or 8% (HS) to induce prehypertension. Each batch was divided into two group and treated by bilateral PVN microinjection with either artificial cerebrospinal fluid or puerarin through a micro-osmotic pump for 6 weeks. The mean arterial pressure (MAP) was recorded, and samples were collected and analyzed. Results: We concluded that puerarin significantly prevented the elevation of blood pressure and effectively alleviated the increase in heart rate caused by high salt. Norepinephrine (NE) in the plasma of salt-induced prehypertensive rats also decreased upon puerarin chronic infusion. Additionally, analysis of the PVN sample revealed that puerarin pretreatment decreased the positive cells and gene level of TLR4 (Toll-like receptor 4), NLRP3, Caspase-1 p10, NOX2, MyD88, NOX4, and proinflammatory cytokines in the PVN. Puerarin pretreatment also decreased NF-κBp65 activity, inhibited oxidative stress, and alleviated inflammatory responses in the PVN. Conclusion: We conclude that puerarin alleviated blood pressure via inhibition of the ROS/TLR4/NLRP3 inflammasome signaling pathway in the PVN, suggesting the therapeutic potential of puerarin in the prevention of hypertension.

## 1. Introduction

Epidemiological data on hypertension revealed that this non-communicable condition affects more than 1 billion adults worldwide [1]. Hypertension is a high-risk factor for cardiovascular and cerebrovascular diseases and significantly increases the incidence of complications such as stroke, coronary heart disease, heart failure, and end-stage renal disease [2]. Due to its complications, hypertension remains an important threat for health systems and a heavy burden for patients, requiring lifetime medication. Different interventions, such as change in lifestyle, exercise, and pharmacologic agents, are used to reduce the signs of hypertension. Once diagnosed with hypertension, medicinal intake remains popular and more effective for reducing blood pressure values without complete eradication. Therefore, preventive measures for people at risk of hypertension may prevent and slow down its onset and development.

A high intake of sodium chloride (salt) constitutes an important risk factor for primary hypertension, and individuals within the population exhibit different blood pressure responses to salt load, suggesting the existence of salt sensitivity within the population. Salt-sensitive hypertensive rats are an ideal animal model for studying salt-induced hypertension and elucidating the mechanisms of preventive and curative interventions. Increasing data support the role of natural drugs in the prevention and treatment of hypertension. Data from both traditional medicine and modern pharmacology support the effectiveness of natural drug compounds for managing hypertension with less side effects, which has attracted the attention of the pharmaceutical industry [3,4].

Despite intensive investigations on hypertension, identifying several mechanisms related to its pathogenesis, the exact cause of this multifactorial condition is still unclear. Extensive research has shown that continuous immune system activation is associated with various types of hypertension [5,6]. The activation of innate immunity, inflammation, and acquired immunity can cause damage and dysfunction of the vascular endothelium, leading to hypertension and its complications, such as coronary heart disease, heart failure, stroke, and chronic kidney disease [7]. Clinical studies have reported a positive correlation between the levels of inflammatory mediators and blood pressure [8]. Also, increased inflammatory cytokines in the central nervous system (CNS) reportedly promotes the pathological development of hypertension [9].

The paraventricular nucleus of the hypothalamus (PVN) is a small region located on the dorsal side of the hypothalamus, on the upper border of the third ventricle, and contains a group of nerve cells with remarkable chemical specificity. The PVN receives various incoming information that reflects the external chemical environment (such as nitric oxide) and can regulate the secretion of inflammatory factors [10]. This information is received through the hypothalamus and periventricular organs, as well as a portion through the afferent nerves and solitary tract nucleus in the vagus nerve [11]. Therefore, the PVN is a heterogeneous nucleus composed of different neurons, playing an important role in regulating autonomic nervous system functions, such as the drive of the sympathetic nervous system [12]. A number of experimental data suggest that a reduction in inflammation in the PVN could improve hypertension. Blocking TNF α and NLRP3 in the PVN can significantly improve blood pressure [13,14]. Additionally, previous studies have shown that long-term regular exercise can reduce the level of inflammation in the PVN, thereby attenuating hypertension in rats [15]. Moreover, Ying Li et al. found that exercise and food supplementation with vitamin C can downregulate PIC levels and upregulate AIC levels in the PVN, improving the inflammatory state of the PVN, and thus having an anti-hypertensive effect [16]. TLR4 is an initiator protein of the body’s inflammatory response chain. Increasing evidence indicates that TLR4/NF-κB signal transduction in the PVN plays a critical role in many animal models of cardiovascular disease, ischemic brain injury, diabetes, atherosclerosis, and other inflammatory diseases [17,18], suggesting the therapeutic potential of targeting this signaling pathway for hypertension. Moreover, previous data suggest that inflammation promotes hypertension by increasing reactive oxygen species (ROS) levels, which leads to oxidative stress [19]. The ROS (such as hydrogen peroxide and superoxide) released by immune cells through NADPH oxidase to attack and protect the body from invading organisms is a physiological process. However, in a number of pathologies, the ROS production becomes excessive and promotes damage. Therefore, blocking oxidative stress can minimize systemic inflammatory responses and alleviate the severity of cardiovascular disease [20]. 

Traditional Chinese medicine is used in treating high blood pressure and can prevent end-organ damage and other complications [21]. Puerarin is a stable isoflavone compound extracted and isolated from the roots of a leguminous plant, the wild kudzu, with various biological activities including anti-inflammatory effects [22,23]. Research has found that it has various functional activities in multiple diseases. In China and Eastern Asia, kudzu has a long history as a nutritional food and a Chinese herbal medicine for treating cardiovascular and cerebrovascular diseases [24]. Puerarin can cross the blood–brain barrier and exert its anti-inflammatory effects in numerous animal models of diseases [25]. In light of previous studies, the current study was designed to assess the preventive effects of puerarin on the onset and progression of hypertension and to verify the hypothesis that puerarin alleviates blood pressure by inhibiting the ROS/TLR4/NLRP3 inflammasome signaling pathway in the PVN of salt-induced prehypertensive rats.

## 2. Materials and Methods 

### 2.1. Animals

The present study was conducted according to the “Guide for the Care and Use of Laboratory Animals” published by the National Institutes of Health (NIH). The use and care of animals in this study were approved by the Laboratory Animal Management Committee of Xi’an Jiaotong University, Xi’an, China (No. 2022-1116). Adult male seven-week-old male Dahl salt-sensitive rats (Beijing Vital River Laboratory Animal Technology Co., Ltd., Beijing, China) weighing between 180 and 210 g were used in this study. They were divided into control groups and model groups based on diet. All animals were placed in separate cages and raised in temperature- and humidity-controlled animal rooms and underwent a 12 h/12 h light and dark cycle. All rats were fed freely with tap water.

### 2.2. General Experimental Protocol 

After one week of adaptive feeding, Dahl salt-sensitive rats were randomly divided into 4 groups based on their diet (0.3% NaCl (NS) or 8% NaCl (HS)) and treatment (puerarin or vehicle injection in the PVN). The distribution of animals was carried out prior to any treatment. The following groups were set: 

I. Control group 1 (NS + PVN vehicle): Dahl salt-sensitive rats (0.3% NaCl) were treated with bilateral PVN infusion of vehicle as negative controls.

II. Control group 2 (NS + PVN puerarin): Dahl salt-sensitive rats (0.3% NaCl) were treated with bilateral PVN infusion of puerarin and regarded as the normal blood pressure treatment group.

III. Experimental group 3 (HS + PVN vehicle): Dahl salt-sensitive rats (high-salt diet, 8% NaCl) were treated with bilateral PVN infusion of vehicle and included in the study as prehypertensive rats.

IV. Experimental group 4 (HS + PVN puerarin): Dahl salt-sensitive rats (high-salt diet, 8% NaCl) were treated with bilateral PVN infusion of puerarin and regarded as the prehypertension treatment group.

Puerarin (Sigma, St. Louis, MO, USA) was administered through chronic bilateral PVN administration by a minipump (Alzet Osmotic Pumps, Model 2006, 0.15 μL/h, Alzet Corp., Cupertino, CA, USA) throughout the animal experiment phase of the study that lasted 6 weeks. We used a non-invasive tail artery blood pressure measurement and analysis system to record changes in blood pressure and heart rate. At the end of the experiment, the rats were anaesthetized and then decapitated or perfused to collect brain tissue, which was processed and preserved.

### 2.3. Mean Arterial Pressure (MAP) Measurement

In this study, we used the non-invasive tail-cuff method to monitor blood pressure. Animals were familiarized with the measurement system for a week in advance. Blood pressure was monitored at a fixed time in the morning from 8:00 to 10:00. Blood pressure and heart rate were recorded once a week, eight times during each recording, and the mean value was calculated. The details of the specific monitoring method are consistent with the previous description [26,27].

### 2.4. Collection of Tissue Samples

Brain tissue was either snap frozen or fixed and processed for immunofluorescence staining. Subsequently, frozen sections were used to detect changes in signal pathway positive cells using immunofluorescence technology. The PVN tissue was isolated following Palkovits’s microdissection procedure, as previously described [28,29]. Tissue samples were stored at −80 °C until assayed. 

### 2.5. Immunofluorescence (IF) Studies

The positive cell expression of TLR4, MyD88, NLRP3, Caspase-1 p10, and NOX2 in the PVN was detected by IF. Rats were perfused under deep anesthesia with ice-cold phosphate-buffered saline (PBS) followed by 4% paraformaldehyde (PFA). The brain was removed and immersed in 4% PFA. After 36 h, brain tissues were dehydrated twice with sucrose until they sank to the bottom. Tissues were thick sectioned (4 mm), embedded in wax with OTC, and sectioned at 14–16 μm. To perform IF, sections were incubated with different primary antibodies overnight, then with the corresponding second antibodies the next day, and the cell nucleus was further stained with 4, 6-diamino-2-phenylindole (DAPI). The following primary antibodies were used: TLR4, MyD88, NLRP3, Caspase-1 p10, and NOX2 in the PVN was detected by IF. For each rat, double-blind manual counting of positive cells within the bilateral boundaries of PVN in three consecutive sections was performed, and the average value was made. The IF protocol was used, as previously described [30,31].

### 2.6. PCR Analysis

Rat brains were isolated and cut into a coronal segment (−0.92 to −2.13 mm posterior to bregma). From the coronal section, we excised a block of the hypothalamus containing the PVN. The primer sequences of TLR4, MyD88, SOD, IL-1β, IL-6, TNFα, and GAPDH are provided in Table 1. The procedure of RT-PCR was the same as the previous research. Total RNA isolation, cDNA synthesis, and RT-PCR were performed as previously described [32,33]. 

### 2.7. ELISA

NE levels were measured in the serum samples using an ELISA kit (H096, Nanjing Jiancheng, Nanjing, China), according to the manufacturer’s instructions.

### 2.8. Oxidative Stress Analysis

Frozen brain sections were incubated with a dihydroethidium (DHE) fluorescent probe. Under the presence of ROS in tissues, fluorescent probes are oxidized to generate red fluorescent substances. The intensity of red fluorescence is directly proportional to the level of ROS in tissues. By detecting the fluorescence of DHE products, the level of reactive oxygen species in tissues can be determined. The intensity of fluorescence was analyzed using Image J 6.0 software [27].

### 2.9. Statistical Analysis

The results were analyzed using GraphPad Prism 8.0 software, and data were expressed as the means ± SEM. Comparisons between multiple groups were performed by two-way ANOVA with Tukey’s multiple comparison tests. MAP data were analyzed using repeated-measures ANOVA. All the data have been tested for normality and homogeneity of variance, and those of image analysis were processed in a blinded fashion. *p*-values < 0.05 were regarded as a statistically significant difference.

## 3. Results

### 3.1. Puerarin Attenuated Blood Pressure 

**Figure 1:**
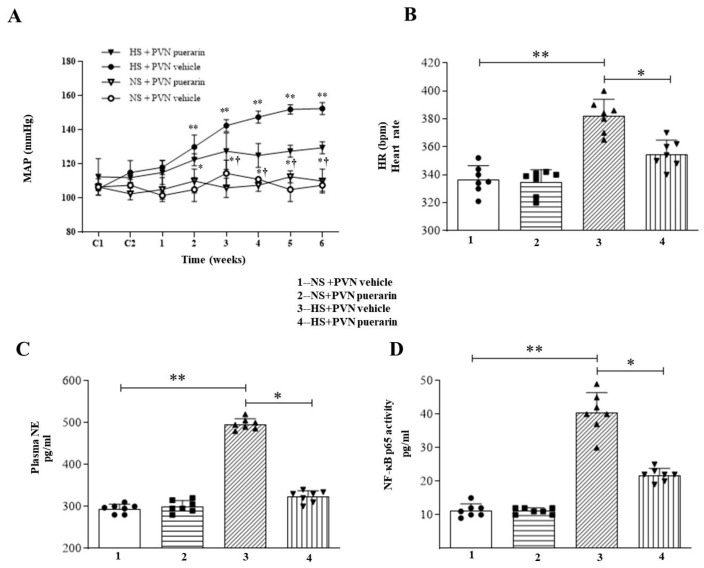
Puerarin treatment ameliorated (**A**) MAP, (**B**) HR, (**C**) NE in plasma levels and (**D**) NF-κB p65 activity in the PVN of salt-induced prehypertension. Non-invasive blood pressure measurement was used to detect the changes of mean blood pressure and heart rate of rats in each group. ELISA kits were used to evaluate the levels of NE in plasma and NF-κB p65 activity in the PVN. The data are expressed as the means ± SEM (*n* = 7 rats/group). * *p* < 0.05, ** *p* < 0.01 vs. control groups; ^†^ *p* < 0.05 vs. puerarin-pretreated groups.

The primary outcome of this study is changing blood pressure (MAP). High salt (8%) intake increased MAP after one week and further increased it throughout the experimental period. Similarly, high salt (8%) intake increased heart rate. Interestingly, PVN infusion of puerarin could prevent high salt (8%) intake-increased MAP and HR (Figure 1A,B). In addition, we used an ELISA kit to detect NE levels in peripheral blood and NF-κB activity in the PVN. As shown in Figure 1C,D, PVN infusion of puerarin decreased high salt (8%)-increased plasmatic NE and NF-κB activity in the PVN. However, PVN infusion of puerarin in the NS group did not affect MAP, HR, NE, and NF-κB activity in the PVN.

### 3.2. Regulatory Effect of Puerarin Treatment on the Expression Levels of TLR4

**Figure 2:**
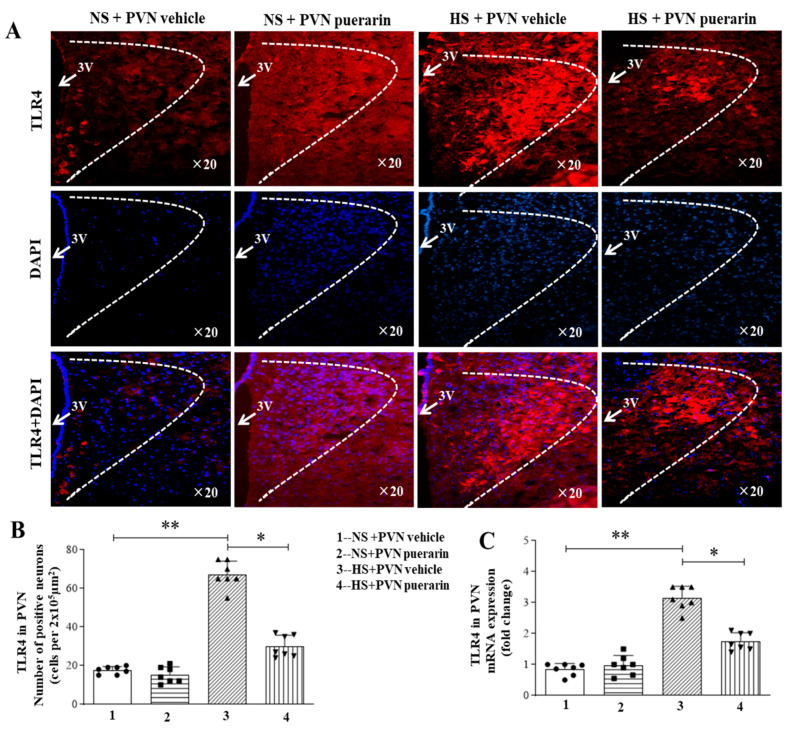
Puerarin treatment ameliorated the number of positive cells of TLR4 in the PVN of salt-induced prehypertension. (**A**) Representative immunofluorescence staining of TLR4 (TLR4: red fluorescence, DAPI: blue fluorescence). (**B**) Densitometric analysis of immunofluorescence staining of TLR4. (**C**) mRNA expression of TLR4.The data are expressed as the means ± SEM (*n* = 7 rats/group). * *p* < 0.05, ** *p* < 0.01. 3V, third ventricle.

In recent years, the anti-inflammatory effect of puerarin has gradually been recognized by people, especially as research on the inflammatory response of cardiovascular diseases becomes more extensive. Therefore, this study used immunofluorescence and PCR techniques to detect relevant indicators of the TLR signaling pathway. As shown in Figure 2, there was an increased number of TLR4 positive cells in response to high salt (8%) intake in the PVN, as illustrated by immunofluorescence representative microphotographs (Figure 2A) and cell count graphs (Figure 2B). This was also associated with increased TLR4 gene expression levels (Figure 2C) in the HS + PVN vehicle group. However, PVN infusion of puerarin significantly decreased the number of positive cells and the gene expression level of TLR4 in the PVN in prehypertensive rats. Moreover, the PVN infusion of puerarin in the NS group did not affect the number of TLR4 positive cells and TLR4 gene expression in the PVN.

### 3.3. Regulatory Effect of Puerarin Treatment on the Expression Levels of MyD88

**Figure 3:**
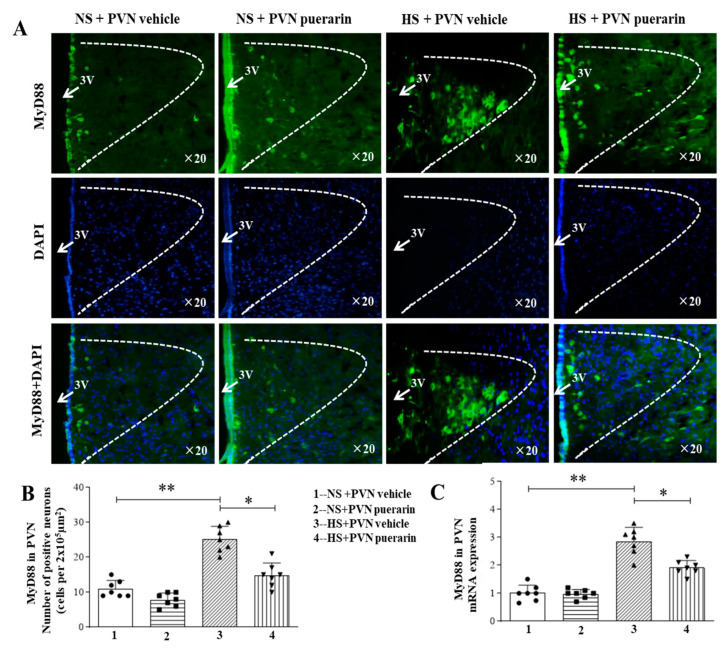
Puerarin treatment ameliorated the number of positive cells of MyD88 in the PVN of salt-induced prehypertension. (**A**) Representative immunofluorescence staining of MyD88 (MyD88: green fluorescence, DAPI: blue fluorescence). (**B**) Densitometric analysis of immunofluorescence staining of MyD88. (**C**) mRNA expression of MyD88. The data are expressed as the means ± SEM (*n* = 7 rats/group). * *p* < 0.05, ** *p* < 0.01. 3V, third ventricle.

As shown in Figure 3A,B, there was an increased number of MyD88 positive cells in response to high salt (8%) intake in the PVN, as illustrated by immunofluorescence representative microphotographs (Figure 3A) and cell count graphs (Figure 3B). This was also associated with increased MyD88 gene expression levels (Figure 3C) in the HS + PVN vehicle group. However, PVN infusion of puerarin significantly decreased the number of positive cells and the gene expression level of MyD88 in the PVN in prehypertensive rats. Moreover, the PVN infusion of puerarin in the NS group did not affect the number of MyD88 positive cells and MyD88 gene expression in the PVN. 

### 3.4. Regulatory Effect of Puerarin Treatment on the Expression Levels of NLRP3

**Figure 4:**
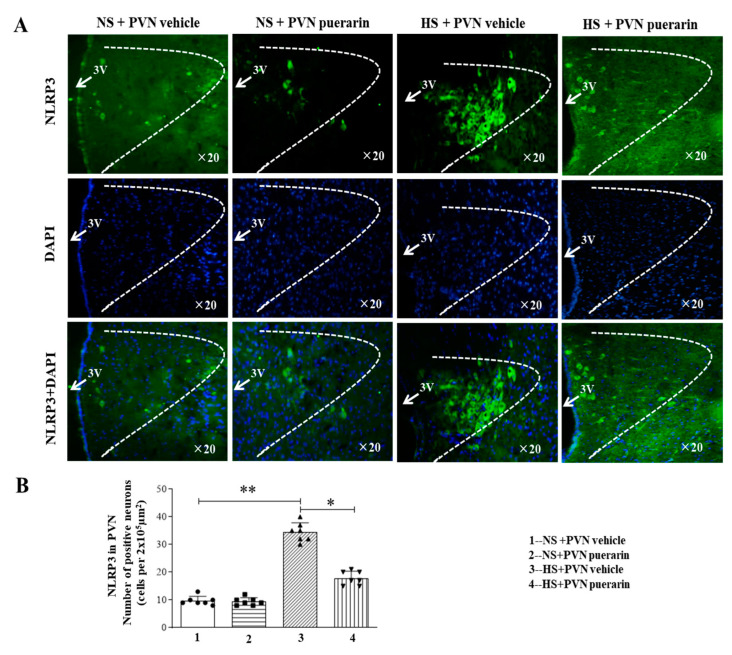
Puerarin treatment ameliorated the number of positive cells of NLRP3 in the PVN of salt-induced prehypertension. (**A**) Representative immunofluorescence staining of NLRP3 (NLRP3: green fluorescence, DAPI: blue fluorescence). (**B**) Densitometric analysis of immunofluorescence staining of NLRP3. The data are expressed as the means ± SEM (*n* = 7 rats/group). * *p* < 0.05, ** *p* < 0.01. 3V, third ventricle.

High salt (8%) intake increased the number of NLRP3 positive cells in the PVN, as illustrated by immunofluorescence representative microphotographs (Figure 4A) and cell count graphs (Figure 4B). However, PVN infusion of puerarin significantly decreased the number of positive cells of NLRP3 in the PVN in prehypertensive rats. Moreover, the PVN infusion of puerarin in the NS group did not affect the number of NLRP3 positive cells in the PVN.

### 3.5. Regulatory Effect of Puerarin Treatment on the Expression Levels of Caspase-1 p10

**Figure 5:**
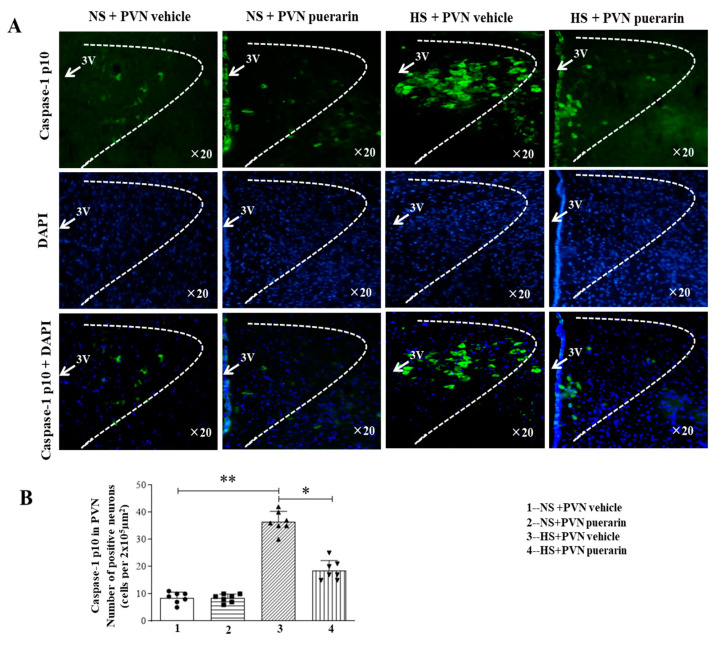
Puerarin treatment ameliorated the number of positive cells of Caspase-1 p10 in the PVN of salt-induced prehypertension. (**A**) Representative immunofluorescence staining of Caspase-1 p10 (Caspase-1 p10: green fluorescence, DAPI: blue fluorescence). (**B**) Densitometric analysis of immunofluorescence staining of Caspase-1 p10. The data are expressed as the means ± SEM (*n* = 7 rats/group). * *p* < 0.05, ** *p* < 0.01. 3V, third ventricle.

High salt (8%) intake increased the number of Caspase-1 p10 positive cells in the PVN, as illustrated by immunofluorescence representative microphotographs (Figure 5A) and cell count graphs (Figure 5B). However, PVN infusion of puerarin significantly decreased the number of positive cells of Caspase-1 p10 in the PVN in prehypertensive rats. Moreover, the PVN infusion of puerarin in the NS group did not affect the number of Caspase-1 p10 positive cells in the PVN.

### 3.6. Regulatory Effect of Puerarin Treatment on the Expression Levels of Oxidative Stress

**Figure 6:**
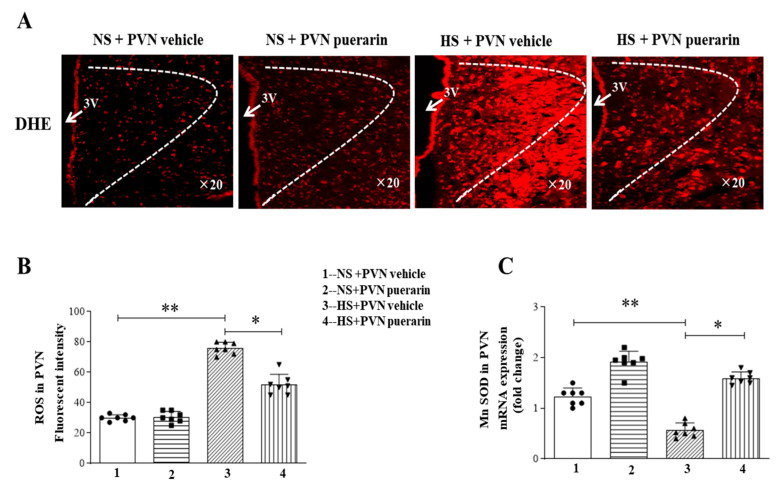
Puerarin treatment ameliorated oxidative stress in the PVN of salt-induced prehypertension. (**A**) Representative microphotographs of DHE staining. (**B**) Densitometric analysis of immunofluorescent intensity of DHE in the PVN. (**C**) mRNA expression of Mn-SOD. The data are expressed as the means ± SEM (*n* = 7 rats/group). * *p* < 0.05, ** *p* < 0.01. 3V, third ventricle.

The imbalance between oxidation and antioxidants is a key factor affecting blood pressure fluctuations. Research has shown that changes in oxidative stress in the PVN can affect sympathetic nervous system activity. Given this, we investigated whether puerarin can exert its antioxidant effects in the PVN, thereby attenuating increased blood pressure. As illustrated in Figure 6A,B, DHE staining showed elevated levels of ROS and the reduced expression level of SOD genes (Figure 6C) in the HS + PVN vehicle group, all of which were remarkably reverted by the PVN infusion of puerarin.

**Figure 7:**
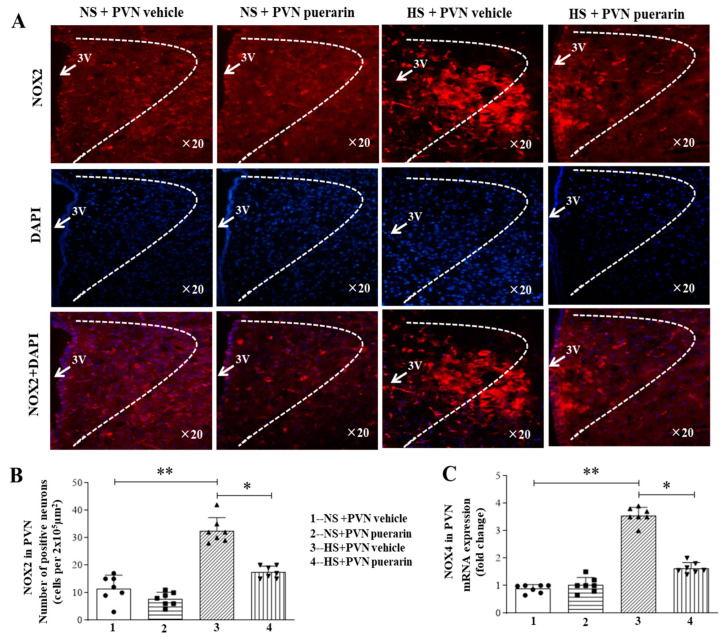
Puerarin treatment ameliorated NAD(P)H oxidase subunit NOX2 and NOX4 in the PVN of salt-induced prehypertension. (**A**) Representative immunofluorescence staining of NOX2 (NOX2: red fluorescence, DAPI: blue fluorescence). (**B**) Densitometric analysis of immunofluorescence staining of NOX2. (**C**) mRNA expression of NOX4. The data are expressed as the means ± SEM (*n* = 7 rats/group). * *p* < 0.05, ** *p* < 0.01. 3V, third ventricle.

This study also used immunofluorescence staining to quantify the positive cell levels of NOX2 and RT-PCR for NOX4 gene expression. There is an increased number of NOX2 positive cells (Figure 7A,B) and NOX4 gene expression (Figure 7C) in response to high salt intake in the HS + PVN vehicle group. However, the PVN infusion of puerarin has significantly decreased the number of NOX2 positive cells and the gene expression level of NOX4 in the PVN (Figure 7A–C).

### 3.7. Puerarin Regulated the Expression Levels of the Inflammatory Cytokine Levels 

**Figure 8:**
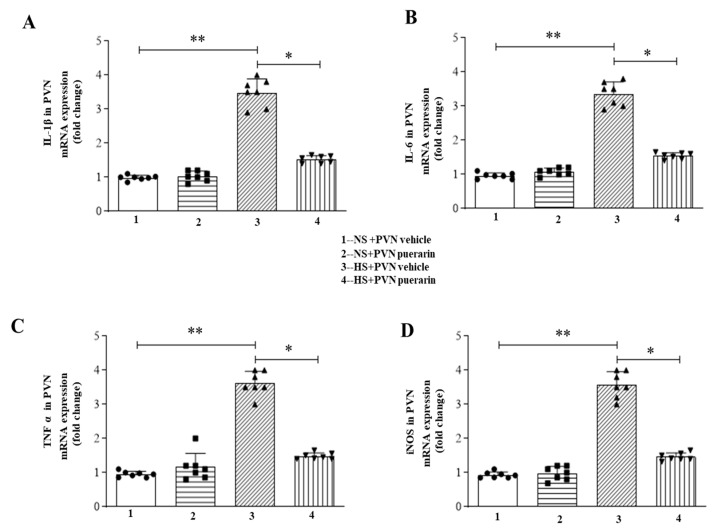
Puerarin pretreatment ameliorated inflammatory cytokines in the PVN of salt-induced prehypertension. (**A**) mRNA expression of NOX4. (**B**) mRNA expression of IL-6. (**C**) mRNA expression of TNF α. (**D**) mRNA expression of iNOS. The data are expressed as the means ± SEM (*n* = 7 rats/group). * *p* < 0.05, ** *p* < 0.01. 3V, third ventricle.

We also evaluated the proinflammatory marker expression in the PVN. We used RT-PCR to detect changes in the expression of these indicators. As shown in Figure 8, PCR analysis revealed that the gene expression levels of IL-1β (Figure 8A), IL-6 (Figure 8B), and TNF-α (Figure 8C) were dramatically increased, and that of IL-10 (Figure 8D) was decreased in the HS + PVN vehicle group. The PVN infusion of puerarin reversed these changes.

## 4. Discussion

Hypertension is a disease with a high mortality and morbidity rate, and has become one of the major causes of death worldwide [34]. Hypertension is currently a lifelong incurable disease and causes non-clarified etiopathogenesis [35] due to the complexity of its pathogenesis and the large individual differences among hypertensive patients. This ultimately set a variety of pharmacological interventions. Puerarin, also known as dihydroxy isoflavones, is isolated from the root of a traditional Chinese medicinal plant, Pueraria lobata, and has a series of beneficial activities for cardiovascular diseases [36,37]. Research has confirmed that puerarin may have a protective effect on blood pressure [38]. However, the mechanisms by which puerarin improves blood pressure remain unclear. The current study showed that pretreatment with puerarin effectively prevented the salt-induced elevation of blood pressure. The findings of this study are summarized as follows: (I) the ROS/TLR4/NLRP3 pathway in the PVN has been involved in promoting the process of blood pressure elevation in prehypertension; (II) puerarin attenuated NADPH oxidase-dependent ROS generation, suggesting it can exert antioxidant effects in the central nervous system; (III) puerarin exerted its anti-inflammatory effect in the PVN, possibly through the TLR4 pathway; (IV) detailed mechanistic studies revealed that puerarin alleviates blood pressure via inhibition of the ROS/TLR4/NLRP3 inflammasome signaling pathway in the hypothalamic paraventricular nucleus of salt-induced prehypertensive rats. Collectively, these results indicate that puerarin is a new candidate treatment for preventing hypertension.

Many studies have shown that prehypertension is an independent risk factor for cardiovascular and cerebrovascular diseases, and as blood pressure increases, the risk of cardiovascular disease increases accordingly [39,40,41]. Therefore, blood pressure management is essential for prehypertensive individuals. At present, the most important issue regarding medication treatment for prehypertension is whether medication treatment is needed for prehypertension. Identifying molecular targets with significant antihypertensive effects, observing the therapeutic effects of antihypertensive drugs on hypertension by intervening in these targets, and providing a scientific experimental and theoretical basis for the prevention and treatment of hypertension is necessary. According to existing research, it is undeniable that antihypertensive drug therapy can bring benefits to patients with prehypertension. Due to fewer side effects, traditional Chinese medicine may be a complementary strategy to address the insufficient treatment of antihypertensive drugs in the early stages of hypertension. Phytomedicine may provide inspiring methods for curing human diseases [42], warranting further research on the treatment of prehypertension with traditional Chinese medicine [43,44].

The role of oxidative stress on the onset and progression of hypertension has been documented [45,46]. Attenuating oxidative stress can alleviate hypertension and its associated end-organ damage, suggesting the therapeutic potential of antioxidants for hypertension [47]. Interestingly, the changes in ROS levels in the PVN directly affect the sympathetic nervous system and, subsequently, the changes in blood pressure [48]. NADPH oxidase is a key enzyme causing an increase in ROS in the cardiovascular system. Previous studies have revealed the neuroprotective effects of puerarin on antioxidants in rats [49,50]. Puerarin has been shown to counteract neuronal damage induced by cerebral ischemia-reperfusion, possibly by activating Nrf2 through PI3K/Akt and promoting downstream antioxidant enzyme expression, thereby further improving oxidative stress [50]. In addition, puerarin has been revealed to improve neuronal damage in rats by reducing Nrf2-mediated oxidative stress [51]. In this study, we used the reactive oxygen species (ROS) detection kit (DHE) to measure ROS levels in the PVN and found that puerarin treatment can reduce ROS levels in salt-induced prehypertensive rat PVN and increase SOD levels. Moreover, puerarin clearly reduced the levels of NOX2 and NOX4 in the PVN. These findings indicate that puerarin can inhibit oxidative stress through its antioxidant effect in the PVN.

Currently, the concept of hypertension as a low-grade inflammatory state is well documented, as well as the contribution of inflammation to the onset and development of hypertension and target organ damage [7,52]. In animals, increased inflammatory cytokines in the PVN promote a rise of blood pressure [33,53]. Interestingly, puerarin reportedly has anti-inflammatory and neuroprotective effects [54] in both central and peripheral nervous system neurological disorders [55]. Such protective effects involve the inhibition of nervous system inflammation, the regulation of multiple nerve injury pathways, the reduction in apoptosis factor expression, and regulation of the expression of related proteins [56,57]. Studies have shown puerarin could improve renin-angiotensin system (RAS) components, which is intricately involved in maintaining blood pressure homeostasis. Puerarin may attenuate Ang II-induced inflammation in other diseases. Puerarin treatment may ameliorate inflammation via the RAS and the NF-κB pathway in gunpowder acute lung injury [58]. In addition, puerarin is also a Chinese herbal remedy used to prevent and treat atherosclerosis by inhibiting inflammasome activation [59,60]. Our data showed that pretreatment of rats with puerarin significantly reduced the TLR4/NLRP3 inflammasome signaling pathway in the PVN, thereby regulating blood pressure.

Finally, our study has some limitations. Firstly, this study explored the effect of puerarin only in high salt-induced hypertension despite the existence of a variety of hypertensive models, which involve different pathophysiological processes. Secondly, as the immune resident cells in the CNS, emphasis on the behavior of microglia as a neuroinflammatory mediator was not made. Therefore, our next work plan is to use a combination drug delivery system at the cellular level to study its mechanism.

Taken together, our results elucidated that PVN pretreatment puerarin could alleviate the inflammatory response and Nrf2-mediated intracellular ROS, which mediate sympathetic excitability and elevated arterial pressure. We first revealed the central mechanism of the early prevention of high salt-induced hypertension by puerarin. Puerarin alleviates blood pressure via inhibition of the ROS/TLR4/NLRP3 inflammasome signaling pathway in the PVN of salt-induced prehypertensive rats. The findings of the current study provided a research basis for exploring potential new functions of puerarin in the prevention of hypertension.

## 5. Conclusions

**Figure 9:**
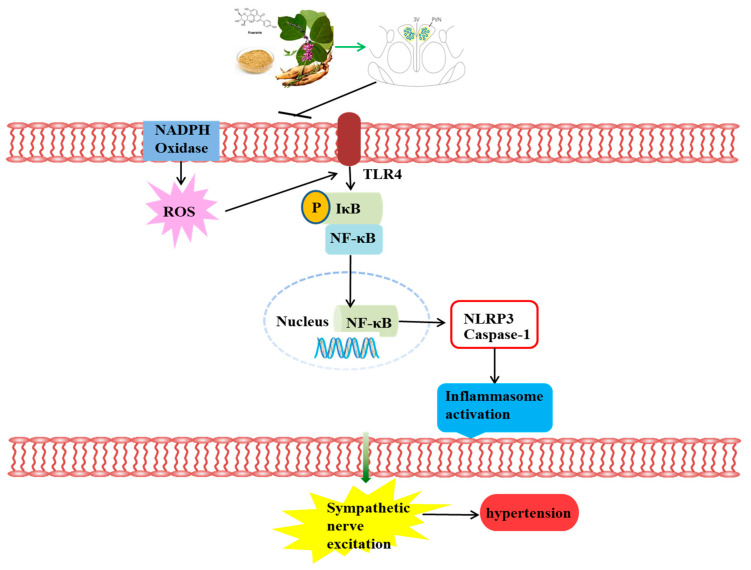
A schematic diagram displaying the effect of puerarin on blood pressure. Puerarin improved salt-induced prehypertension, which may occur via the ROS/TLR4/NLRP3 inflammasome signaling pathway in the PVN.

In summary, the results of this study indicate that puerarin attenuates blood pressure in response to high salt intake in rats. Such an effect may involve the inhibition of the ROS/TLR4/NLRP3 inflammasome signaling pathway in the PVN of salt-induced prehypertensive rats (Figure 9). This suggests that puerarin is a promising drug for preventing hypertension.

## Figures and Tables

**Table 1:** Rat primers used for real-time RT-PCR.

Rat Genes	Sense	Antisense
TLR4	GGCTGTGGAGACAAAAATGACCTC	AGGCTTGGGCTTGAATGGAGTC
MyD88	TCAACAAGCGAGCGCACCGT	TGAGCGCGACCAACGGTAGA
NOX4	TTGGCTGTCCCTAAATGTCC	GCTCTGCTCAAACACAATCCT
MnSOD	ACCTGCCTTACGACTATGG	CCAGTTGATTACATTCCAAAT
iNOS	CCTTGTTCAGCTACGCCTTC	GGTAGCCCGAGTTCTTTCA
MCP-1	GTGCTGACCCCAATAAGGAA	TGAGGTGGTTGTGGAAAAGA
IL-1β	GCAATGGTCGGGACATAGTT	AGACCTGACTTGGCAGAGGA
IL-6	TCTCTCCGCAAGAGACTTCCA	ATACTGGTCTGTTGTGGGTGG
TNF α	ACCACGCTCTTCTGTCTACTG	CTTGGTGGTTTGCTACGAC
GAPDH	AGACAGCCGCATCTTCTTGT	CTTGCCGTGGGTAGAGTCAT

## Data Availability

The original contributions presented in the study are included in the article, further inquiries can be directed to the corresponding author.

## Abbreviations

aCSF	artificial cerebrospinal fluid
AIC	anti-inflammatory cytokines
CNS	central nervous system
DHE	dihydroethidium
IL-10	interleukin 10
IL-1β	interleukin 1β
IL-6	interleukin 6
MAP	mean arterial pressure
MyD88	myeloid differentiation factor 88
NE	norepinephrine
NF-κB	nuclear factor-kappa B
Nrf2	nuclear transcription related factor-2
PBS	phosphate-buffered saline
PICs	proinflammatory cytokines
PVN	paraventricular nucleus
ROS	reactive oxygen species
RAS	renin-angiotensin system
TLR4	Toll-like receptor 4
TNF α	tumor necrosis factor α
